# Targeting EZH2 in Cancer: Mechanisms, Pathways, and Therapeutic Potential

**DOI:** 10.3390/molecules29245817

**Published:** 2024-12-10

**Authors:** Maria Saveria Gilardini Montani, Rossella Benedetti, Mara Cirone

**Affiliations:** Department of Experimental Medicine, Sapienza University of Rome, 00161 Rome, Italy; rossella.benedetti@uniroma1.it

**Keywords:** EZH2, methylation, oncogenic pathways, immune escape and EZH2 inhibitors

## Abstract

Enhancer of zeste homolog 2 (EZH2) is a methyltransferase involved in cell cycle regulation, cell differentiation, and cell death and plays a role in modulating the immune response. Although it mainly functions by catalyzing the tri-methylation of H3 histone on K27 (H3K27), to inhibit the transcription of target genes, EZH2 can directly methylate several transcription factors or form complexes with them, regulating their functions. EZH2 expression/activity is often dysregulated in cancer, contributing to carcinogenesis and immune escape, thereby representing an important target in anti-cancer therapy. This review summarizes some of the mechanisms through which EZH2 regulates the expression and function of tumor suppressor genes and oncogenic molecules such as STAT3, mutant p53, and c-Myc and how it modulates the anti-cancer immune response. The influence of posttranslational modifications on EZH2 activity and stability and the possible strategies leading to its inhibition are also reviewed.

## 1. Introduction

**Figure 1:**
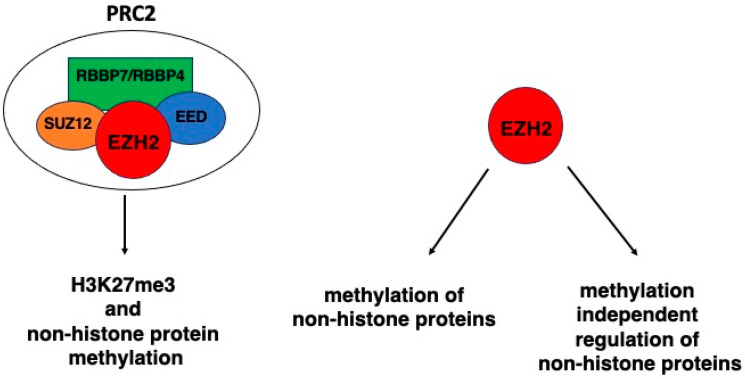
EZH2 activities on H3 histone and non-histone proteins dependent or not on PRC2.

Histones are the core component of the nucleosome, which is the basic unit of chromatin. Histone modifications form readable codes that regulate transcriptional activation and gene silencing. Among the posttranslational modifications to which histones may undergo, a key role is played by methylation, a process that involves the activity of several methyltransferases and de-methylases and may result in the repression or activation of transcription, depending on the residues and the entity of methylation [1]. One of the most important methyltransferases is Enhancer of zeste homolog 2 (EZH2), a SET domain-containing protein which classically acts in the context of Polycomb repressive complex 2 (PRC2). The EZH2 gene, located on chromosome 7q35, consists of 20 exons encoding a protein with 746 amino acid residues [2]. The Polycomb group (PcG) is a family of negative regulators of gene transcription that comprises two distinct complexes with different enzymatic activities: PRC1 with histone ubiquitin ligase activity and PRC2 with histone methyltransferase activity [3]. The catalytic activity of PRC2 relies on the presence and integrity of its four core subunits that, besides EZH2 or related EZH1, comprise embryonic ectoderm development (EED), suppressor of zeste 12 homolog (SUZ12), and retinoblastoma-binding protein 7 (RBBP7) or related RBBP4 (Figure 1). It is known that the EZH2 subunit must be complexed with SUZ12 and EED, to attain robust histone methyltransferase activity [4]. Other accessory units that regulate PRC2 enzymatic activity and function include AEBP2, PCLs, and JARID2. The SET domain of EZH2 is a highly conserved domain, found in chromatin-associated regulators of gene expression, representing the methyltransferase active site of the protein [5]. Notably, EZH2 can also exert its methyltransferase activity on several non-histone proteins dependently or independently of PRC2 and may regulate them dependently or independently of methylation (Figure 1).

## 2. EZH2 and H3K27 and Tumor Suppressor Genes

Working coordinately with the other PcG proteins in the PRC2, EZH2 primarily induces trimethylation of histone H3 on lysine 27 (H3K27me3), resulting in the silencing of gene transcription [6,7,8,9]. This activity represents the canonical function of EZH2, which is responsible for developmental patterning, X chromosome inactivation, stem cell maintenance and cell-fate decision [3,10,11,12]. Interestingly, in several pathological contexts, EZH2 may also play non-canonical functions and work independently of PRC2. As an epigenetic repressor, for example, EZH2 may, in some cases, contribute to H3K9 tri-methylation, a histone target that is primarily methylated by the methyltransferase G9a [13]. EZH2 mutations and abnormal expression have been implicated in the onset and progression of cancer [14]. For example, it has been recently highlighted that mutations in the SET domain of EZH2 in human lymphomas result in aberrant activation of PcD and elevation of H3K27me3 [15,16,17]. Heterozygous point mutations of EZH2 occurring at tyrosine 641 (Y641) within the C-terminal catalytic SET domain of EZH2 are observed in 22% of germinal center B-cell (GCB) diffuse large B-cell lymphomas (DLBCL) and 7%—12% of follicular lymphomas (FL), and contribute to EZH2’s oncogenic potential. EZH2 overexpression was demonstrated for the first time in the context of prostate cancer [18] and later on observed in many other solid tumors such as breast, lung, hepatocellular, colorectal, and pancreatic cancers [19], as well as hematological cancers [20]. EZH2 expression has also been correlated with the aggressiveness and metastasis capacity of breast and prostate cancers [21,22].

EZH2 promotes carcinogenesis mainly by repressing tumor suppressor genes, through the trimethylation of H3K27. For example, onco-suppressors such as p21 and p16 may be activated by EZH2 depletion, independently of p53 [23]. Moreover, the loss of PSP94, a prostatic secretory protein of 94 amino acids, acting as a tumor suppressor in advanced hormone-refractory prostate cancer, may occur in correlation with an increased expression of EZH2, via the trimethylation of histone H3K27, in the MSMB gene, which encodes for PSP94 [24]. EZH2 can also collaborate with DNA methyltransferases (DNMTs) to reinforce gene silencing through DNA methylation and Polycomb group targets are more likely to have cancer-specific promoter DNA hypermethylation compared to non-targets [25].

**Figure 2:**
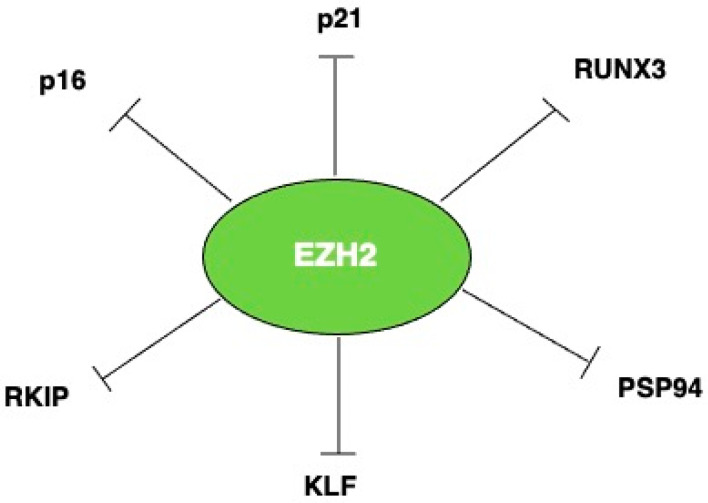
Molecules inhibited by EZH2 in an H3K27-dependent manner.

Moreover, an inverse correlation between EZH2 and RUNX3 expression has been shown to be dependent on the trimethylation of histone H3K27 on RUNX3 gene promoter, in several cancer cell lines [26]. The Kruppel-like factor (KLF) protein 2 epigenetic silencing also occurs in correlation with H3K27 trimethylation induced by EZH2 [27]. The negative regulation of the transcription of the tumor and metastasis suppressor Raf-1 kinase inhibitor protein (RKIP) is also mediated by this repressive histone modification, in breast as well as in prostate cancer cells [28] (Figure 2).

## 3. EZH2 and Non-Histone Protein Methylation: The Case of GATA4, RORα, and PLZF

**Figure 3:**
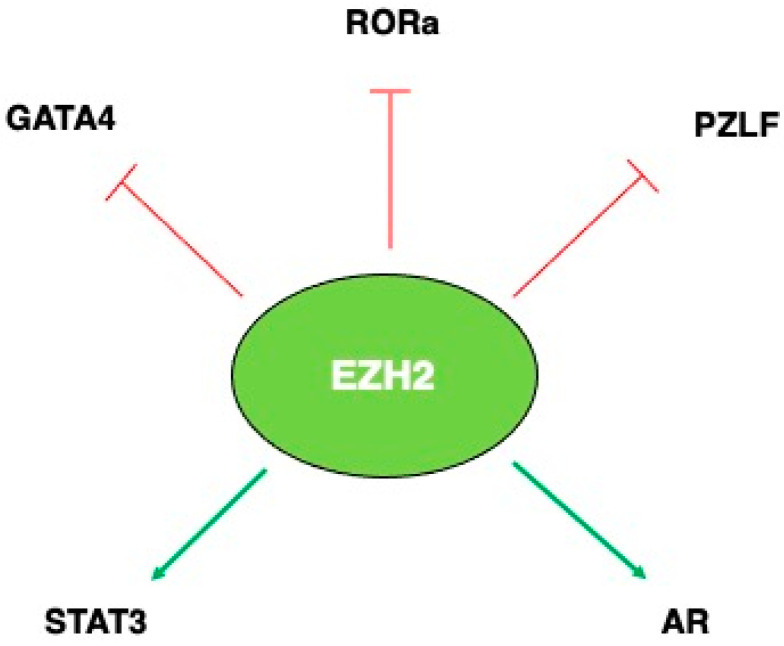
Molecules regulated by EZH2 by direct protein methylation.

Intriguingly, EZH2 can behave as an activator or repressor of gene expression [29] and act independently of H3 methylation, by inducing the methylation of some non-histone proteins or by directly interacting with other proteins, both in a PCR2-dependent (PcD) and PRC2-independent (PcI) manner. EZH2 has been reported to methylate transcription factors (TFs) such as GATA4, RORα, PLZF, AR, and STAT3, with a strong impact on their activities (Figure 3). In this regard, He et al., demonstrated for the first time that PRC2/EZH2 directly methylates the transcription factor GATA4, a key regulator of heart development in mice and humans, resulting in an attenuation of its transcriptional activity. The authors showed that the methylation of GATA4 resulted in a significant inhibition of GATA4 acetylation by the acetyltransferases (HAT) p300. PRC2 represses GATA4 transcriptional activity by methylating the residue K299 and inhibiting the binding of it to p300, as demonstrated by using the GATA4[K299R] mutant (mutant that binds to both p300 and EZH2, but cannot be methylated) [30].

Among the non-histone targets of EZH2, there is the Retinoic Acid-Related Orphan Nuclear Receptor α (RORα), considered a tumor suppressor, as its activation correlates with a reduced migratory and invasiveness of androgen-independent prostate cancer cells [31] and with a reduced canonical Wnt/b-catenin signaling in colon cancer [32]. The authors of the study showed that EZH2 was able to monomethylate RORa, causing a degradation of the protein through the ubiquitin–proteasome pathway, thereby inhibiting its tumor suppressive role. Moreover, an inverse correlation has been observed between the expression of EZH2 and RORa in breast tumor tissues and their normal counterparts, as the RORa protein expression was level very low in tumors exhibiting high levels of EZH2 [33].

As reported for RORa, the methylation and degradation of the Promyelocytic Leukemia Zinc Finger Protein (PLZF) may also be induced by EZH2 [34]. PLZF is an important transcription factor driving the NKT innate-like effector differentiation during thymic development in the NK lineage [35]. In this study, the K430 lysine residue of PLZF was identified as a targeted of EZH2 methylation, and the importance of the enzymatic activity of EZH2 in the regulation of PLZF was demonstrated by using the small molecule inhibitor GSK126.

Differently from the above reported molecules, androgen receptor (AR) methylation by EZH2 results in its activation, although EZH2 needs to be phosphorylated in order to act as transcriptional co-activator in this context [36]. For the interplay between EZH2 and STAT3, a paragraph specifically describing this interaction is appended below.

### EZH2 and STAT3

STAT3 is a transcription factor whose constitutive activation in most of cancers drives cell survival and proliferation, e.g., by promoting the transcription of survivin, cyclinD1 and c-Myc [37]. STAT3 activation in cancers is rarely due to mutations and mainly relies on post-translational modifications. Intriguingly, STAT3 cross-talks with oncogenic molecules such as mutp53, resulting in a reciprocal support [38,39] and cooperates with EZH2 to sustain carcinogenesis [40], and therefore, mutp53, STAT3 activation, and EZH2 hyperexpression are reported to coexist in cancer cells.

A previous study has reported that EZH2, when phosphorylated at serine 21 (S21) by AKT, can bind to STAT3, methylating this transcription factor, primarily at K180 residue, enhancing tyrosine phosphorylation and activity [41]. STAT3 can also be di-methylated at K49 residue by EZH2, resulting in the modulation of IL-6-responsive transcription [42]. The activation of STAT3 by EZH2 methylation has also been observed in the context of breast cancer [43]. Next, the opposite has been observed, as STAT3 signaling can induce the EZH2 transcriptional activation. EZH2 hyper-expression and STAT3 activation are concomitantly observed in the context of gastric cancer with poor prognosis [40]. However, STAT3 activity may also be inhibited when EZH2 is phosphorylated at T372 by protein kinase A (PKA), as it induces a dominant-negative EZH2 phenotype. The enhanced interaction between T372-phosphorylated EZH2 and STAT3 reduced STAT3 phosphorylation, downregulating interleukin 6 receptor expression and inhibiting the cell proliferation and migration of epithelial ovarian cancer cells in vitro and decreasing ovarian xenograft tumor growth in vivo [44]. A recent report from our group has shown that EZH2 inhibition by Valemetostat instead slightly affected STAT3 tyrosine phosphorylation in primary effusion lymphoma, cells in which this transcription factor is constitutively activated [45].

## 4. EZH2 and Non-Histone Protein Regulation Independent on Methylation

**Figure 4:**
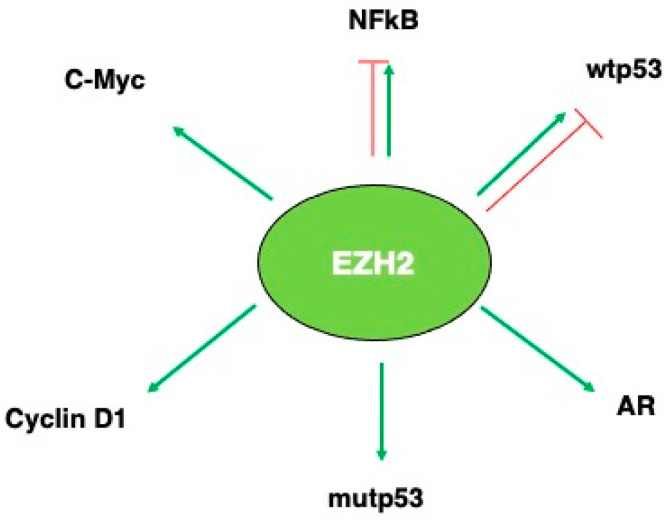
Molecules regulated by EZH2 independently of promoter or protein methylation.

In a PRC2-independent (PcI) and methylation-independent manner, EZH2 may regulate several proteins including NF-kB, androgen receptor (AR) and cyclin D1 (Figure 4). NF-kB was one of the first non-histones identified as target of EZH2 independently on its methylation activity. EZH2 can form a ternary complex with RelB and RelA, promoting the activation of NF-kB and its target genes such as IL-6 and TNF in ER-negative basal-like breast cancer cells. However, EZH2 can behave also oppositely in the regulation of NF-kB target gene expression, depending on the cellular context, as the same authors provided evidence that in the ER-positive luminal-like breast cancer cells, EZH2 negatively regulated NF-kB target genes [46].

Recently, Kim et al. identified another methylation-independent activity of EZH2 in prostate cancer, by discovering that it activates AR gene transcription through direct occupancy of its promoter, in both androgen-dependent prostate cancer (ADPC) and CRPC [47].

EZH2 exerts the PcI effect not only in endocrine-related cancers such as breast and prostate, but also in other aggressive cancers, such as the natural killer/T-cell lymphoma (NKTL). Yan et al. indeed found that EZH2 overexpression in NKTL directly promoted the transcription of cyclin D1, independently of its enzymatic activity [48]. Last but not least, EZH2 can regulate, independently of methylation, transcription factors playing a key role in carcinogenesis such as p53, both wt and mutants, as well as c-Myc, as described in the following sections of this review.

### 4.1. EZH2 and p53

Wild-type (wt)p53 and mutant (mut)p53 may act as oncosuppressor and oncogene, respectively, and posttranslational modifications (PTMs), to which both proteins may undergo, can influence, albeit in an opposite manner, their activity [49]. Among the PTMs, methylation, including that mediated by EZH2, plays an important role in their regulation. It has been observed that EZH2 is overexpressed in cancers carrying p53 mutations [50]. Wtp53 can directly repress EZH2 by binding to the EZH2 promoter [51], as well as indirectly, by upregulating the expression of miR-26a, which represses EZH2. Oppositely to wtp53, mutp53 suppresses miR-26a expression, upregulating EZH2 [52]. Given the oncogenic potential that EZH2 shows in several cancers, its activation by mutp53 it is not surprising and may be included among its so called “gain of function” (GOF). More recently, it has been shown that EZH2 acts as a p53 mRNA-binding protein, binding to IRES1 of wt and mutp53 mRNA and enhancing protein translation in a methyltransferase-independent manner. In the case of tumors harboring p53 GOF mutant, EZH2 cooperates with mutp53 to promote cancer progression [53]. Other authors have reported that targeting EZH2 is a more promising therapeutic strategy against wtp53 carrying cancers, as EZH2 depletion enhances the stability of wt p53 by de-repressing CDKN2A [54]. In line with this study, we have recently reported that the activation of wtp53 contributed to KSHV lytic cycle activation by the EZH2 inhibitor Valemetostat in Primary Effusion Lymphoma cells carrying the virus in a latent state [55]. It has been demonstrated that EZH2 can be ubiquitinated and degraded by the MDM2/MDMX complex. MDM2 is primarily controlled by wtp53, and its expression level is diminished in cancer cells harboring p53 mutations, which may contribute to reduce the turnover of EZH2 and result in the accumulation of EZH2 in mutp53-carrying cells [56]. All these findings suggest that a complex relationship between EZH2 and wtp53 or mutp53 exists, and further studies will shed more light into this promising area of investigation for the treatment of cancer and beyond.

### 4.2. EZH2 and c-Myc

The MYC family contains three members, MYC, MYCN, and MYCL, which encode c-MYC. Human cancers overexpress c-MYC in 70% of cases, due to alterations in signal transduction pathways, such as STAT3 pathway, or due to genomic aberrations, such as amplifications and translocations. Besides being a transcription factor strongly involved in carcinogenesis, c-MYC can modify chromatin structure. It has been reported that by repressing miR-26a, MYC stimulates EZH2 expression in Burkitt lymphoma cells, highlighting how EZH2 deregulation is interconnected with c-MYC-driven tumorigenesis [57]. Moreover, BRD4, a member of BET bromodomain family, may positively regulate EZH2 transcription through the upregulation of c-MYC [58]. EZH2 in turn increases MYCN stabilization independently of its canonical methyltransferase activity. The depletion of EZH2, but not its enzymatic inhibition, induces c-MYC degradation [59]. Again, in a methyltransferase-independent manner, EZH2 can directly bind c-MYC as well as the coactivator (p300), via a cryptic transactivation domain (TAD), inducing gene activation and tumorigenesis. In this study, the authors also evidenced that EZH2 and c-MYC converge to common targets, leading to gene activation, in leukemia.

## 5. EZH2 Posttranslational Modifications

As said above, EZH2 may be regulated by several post-translational modifications (PTMs). PTMs, affecting several proteins and playing a key role in carcinogenesis, have been shown to regulate, either positively or negatively, EZH2 activity. EZH2, similarly to many other proteins, may indeed be decorated with multiple PTMs, occurring in different regions and residues of the molecule [60]. Moreover, as a cross-talk between the different PTMs can occur, and this may further contribute to finely regulate EZH2 stability and activity. Among the PTMs, the most studied is phosphorylation, which may occur on serine (S)/threonine (T) residues. Serine 21 (S21) of EZH2 can be phosphorylated by PI3K/AKT, resulting in an inhibition of its methyltransferase activity in breast cancer [61]. The phosphorylated-EZH2 complex targets crucial nonhistone substrates, for example, S21 phosphorylation of EZH2 enhances EZH2-mediated STAT3 methylation due to a stronger interaction with STAT3 in glioblastoma multiforme (GBM) stem-like cells [41]. In castration-resistant prostate cancer (CRPC), Xu et al. demonstrated that the S21phosphorylation of EZH2 by PI3/AKT switches its ability from gene repressor to transcriptional co-activator of androgen receptor (AR) and AR-associated proteins [36]. EZH2 phosphorylation, occurring on T350 residue, which is catalyzed by CDK1 or CDK2, is required for EZH2-mediated H3K27me3 modifications in prostate cancer cells [62]. Differently from this, the phosphorylation of EZH2 at Y646 residue by JAK2 promotes the β-TrCP-mediated EZH2 degradation [63] and the phosphorylation on Y244 mediated by JAK3 suppresses PRC2 complex formation, resulting in EZH2 oncogenic function in natural killer/T-cell lymphoma [64]. GSK3β also phosphorylates EZH2 on S363, reducing H3K27me3 and attenuating breast cancer oncogenic activity [65]. Moreover, Li et al. demonstrated that AMPK can phosphorylate EZH2 at T311 residue, again inhibiting the EZH2 binding to SUZ12 and resulting in an attenuation of the PRC2-dependent methylation of H3K27 in cancers such as ovarian and breast cancers [66].

Another PTM that affects EZH2 is glycosylation with β-N-acetyl-D-glucosamine (O-GlcNAcylation, GlcNAc). GlcNAcylation at S75 results in the stabilization of EZH2, repressing the expression of important tumor suppression genes [67], while O-GlcNAcylation at the S73, S84, S87, T313, and S729 residues may have several different functions in regulating breast cancer progression [68].

Regarding the acetylation of EZH2, it can be mediated by the acetyltransferase PCAF acetylating the K348 residue of protein, increasing protein stability.

A similar effect has been reported to be induced by (SMYD2) mediating di-methylation at lysine 307 (K307) of EZH2 [69]. However, methylation at K735 site, mediated by SETD2, promotes EZH2 degradation, counteracting prostate cancer metastasis [70]. Notably, EZH2 may methylate itself [71] or other PRC2 accessory cofactors such as JARID2 [72], resulting in an increase in gene repression.

Finally, PRMT1 can mediate the asymmetric di-methylation of EZH2 at R342, which increases its stability and promotes breast cancer metastasis [73]. From this brief overview, it emerges that PTMs may have a strong impact on the stabilization and activity of EZH2 and thus regulate the impact of EZH2 in carcinogenesis.

## 6. EZH2 and Tumor Immune Escape

**Figure 5:**
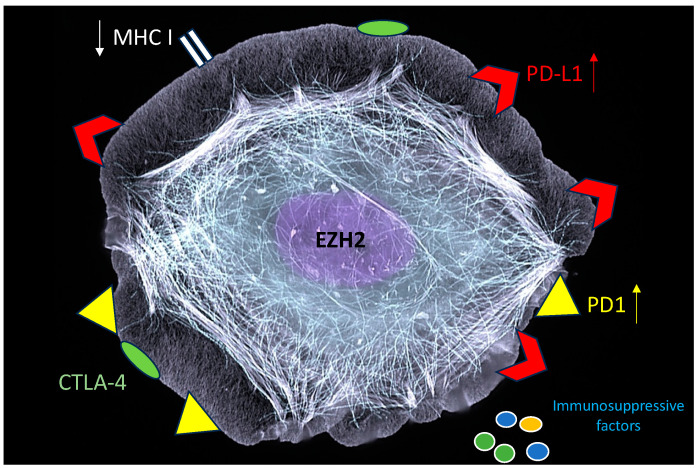
EZH2 dysregulation promotes cancer immune escape.

It is emerging that EZH2 strongly contributes to driving cancer cells’ immunoediting and immune escape [74]. It can interfere by several means with the anti-cancer immune response, e.g., by inducing the upregulation of immune checkpoints such as programmed cell death protein 1 (PD-1), PD-1 ligand 1 (PD-L1), and cytotoxic T-lymphocyte-associated antigen 4 (CTLA-4) [19,75]. Moreover, one of the most important mechanisms leading to cancer immune evasion, namely the suppression of the MHC-class I antigen processing pathway, can be induced by EZH2, favoring an immunosuppressive microenvironment [76]. Through noncanonical mechanisms, EZH2 contributes to the activation of pathways such as JAK/STAT3, which promotes the release of cytokines such as IL-10 and other immune suppressive factors (Figure 5). Moreover, EZH2 upregulation in the cells of tumor microenvironment sustains carcinogenesis, as it promotes the production of pro-angiogenic/immunosuppressive factors by tumor-associated fibroblasts (CAF), suppresses the expression of NKG2D ligands, thus reducing the NK cytotoxic response, promotes the T reg phenotype in T lymphocytes, and alters cellular metabolism increasing the production of lactic acid through the Warburg effect [77,78,79,80]. Last but not least, EZH2 has been reported to increase the recruitment of tumor-associated macrophages (TAMs) by cancer cells overexpressing it [81].

Cancer immunotherapy represents a promising therapeutic option for solid tumors, particularly advanced melanoma, renal cell carcinoma, and non-small-cell lung cancer. However, tumors that better respond to immunotherapies may also develop strategies to resist to it, reducing or abrogating the efficacy of the treatment. Among other mechanisms, epigenetic changes involving EZH2 may drive such resistance. From this evidence, it emerges that targeting EZH2 may be a promising strategy to potentiate and increase the duration of immunotherapies and thus extend the periods of disease-free survival.

## 7. Targeting EZH2 in Cancer Therapy

Selective inhibitors of EZH2 have been developed, and some of them have been approved by the FDA; for example, Tazemetostat has been approved for the treatment of advanced epithelioid sarcoma. EL1, GSK126, CPI-169, EPZ005687, ZLD10A, and GSK503 have been shown to significantly inhibit the H3K27 trimethylation and reduce the survival of different types of lymphomas, while GSK926 and GSK343 have been shown to impair the proliferation of breast cancer cells and prostate cancer cells [82,83].

After inhibiting EZH2, there may be a residual H3K27me3, providing a rationale for using dual EZH2 and EZH1 inhibitors instead of using EZH2 single inhibitors. EZH1/EZH2 dual inhibitors, for example, UNC1999, which is an effective autophagy inducer, has been reported to reduce the diffuse large B-cell lymphoma cell survival [84], and Valemetostat (DS-3201, DS-3201b) is particularly efficient against adult T-cell leukemia lymphoma cells (ATL cells) [85].

Besides targeting the catalytic domain of EZH1 and EZH2, disrupting the protein–protein interactions among the PRC2 subunits is another strategy to inhibit EZH2 activity. In particular, several inhibitors of EZH2-EED interactions have been developed, e.g., the FDA-approved drugs apomorphine hydrochloride, oxyphenbutazone, and nifedipine ergonovine maleate. Others, such as Osimertinib, are already in use for the treatment of metastatic EGFR T790M mutation-positive NSCLC [86]. Moreover, an EED inhibitor has been developed and evaluated in a clinical trial (NCT02900651) for advanced DLBCL, nasopharyngeal carcinoma, gastric cancer, ovarian cancer, prostate cancer, and sarcoma [86].

**Figure 6:**
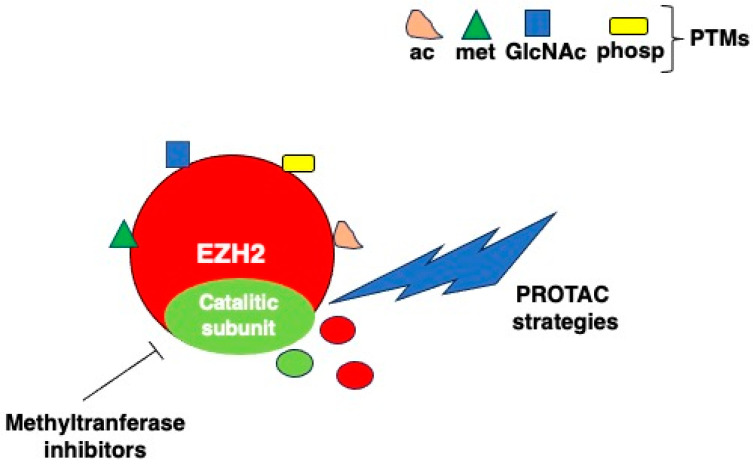
Strategies for targeting EZH2: from posttranslational modifications to enzymatic inhibitors and PROTAC.

As several cancers do not respond to EZH2 enzymatic inhibitors, triggering EZH2 degradation may represent a more efficacious method to inhibit EZH2. As said above, some PTMs can promote EZH2 degradation, mainly through CHIP-mediated ubiquitination [87]. Moreover, EZH2 may be silenced by specific siRNA that reduce its activity (DOI: 10.1155/2014/348728) or its expression may destabilize by miRNAs such as miR-9/9*-124 which can target and repress the expression of USP14, ubiquitin-specific peptidase that stabilizes EZH2 [88]. Finally, given that EZH2 may have non-catalytic oncogenic functions, the enzymatic inhibitors cannot always counteract its activity. Therefore, proteolysis-targeting chimera (PROTAC) degraders have been developed as an alternative therapeutic approach able to inhibit both canonical and non-canonical activity of EZH2. For example, the EZH2 PROTACs MS177 and MS8815 have been reported to be effective in inhibiting the growth of breast cancer cells [89] and PROTAC EZH2 degrader-1 in increasing sensitivity to cisplatin, etoposide, and teniposide in small cell lung cancer (SCLC) [90]. Moreover, compounds such as E7, which can degrade the PRC2 complex, including EZH2, EED, SUZ12, and RbAp48, have been shown to efficiently inhibit EZH2 (Figure 6).

**Figure 7:**
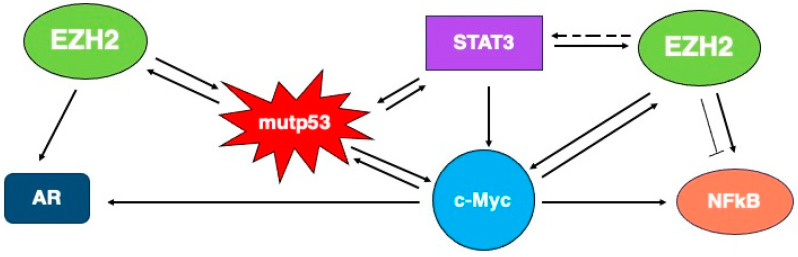
Cross-talks among pro-oncogenic molecules and EZH2.

In conclusion, aberrant EZH2 activity is also emerging as an important player in cancer because, besides contributing to repress the transcription of several tumor suppressor genes, it is interconnected with several oncogenic molecules that regulate and can be regulated by this methyltransferase, both in a methylation-dependent or independent fashion (Figure 7). This suggests that the search for more effective strategies to tune its activity is warranted to improve the outcome of cancer treatment.

## Data Availability

No new data were created or analyzed in this study.
